# Magnetic Response Dependence of ZnO Based Thin Films on Ag Doping and Processing Architecture

**DOI:** 10.3390/ma13132907

**Published:** 2020-06-29

**Authors:** João G. S. Santos, Marcio A. Correa, Armando Ferreira, Bruno R. Carvalho, Rodolfo B. da Silva, Felipe Bohn, Senendxu Lanceiros-Méndez, Filipe Vaz

**Affiliations:** 1 Departamento de Física, Universidade Federal do Rio Grande do Norte, 59078-900 Natal, RN, Brazil; joao.gutavo@gmail.com (J.G.S.S.); marciocorrea@fisica.ufrn.br (M.A.C.); brunorc@fisica.ufrn.br (B.R.C.); rodolfo.bezerra.silva@gmail.com (R.B.d.S.); felipebohn@fisica.ufrn.br (F.B.); 2 Centro de Física, Universidade do Minho, 4710-057 Braga, Portugal; armando.f@fisica.uminho.pt (A.F.); senentxu.lanceros@bcmaterials.net (S.L.-M.); 3 IKERBASQUE, Basque Foundation for Science, E-48013 Bilbao, Spain; 4 BCMaterials, Basque Center for Materials, Applications and Nanostructures, UPV/EHU Science Park, 48940 Leioa, Spain

**Keywords:** zinc oxide, thin films, room temperature ferromagnetism, doping, silver, sputtering

## Abstract

Multifunctional and multiresponsive thin films are playing an increasing role in modern technology. This work reports a study on the magnetic properties of ZnO and Ag-doped ZnO semiconducting films prepared with a zigzag-like columnar architecture and their correlation with the processing conditions. The films were grown through Glancing Angle Deposition (GLAD) co-sputtering technique to improve the induced ferromagnetism at room temperature. Structural and morphological characterizations have been performed and correlated with the paramagnetic resonance measurements, which demonstrate the existence of vacancies in both as-cast and annealed films. The magnetic measurements reveal changes in the magnetic order of both ZnO and Ag-doped ZnO films with increasing temperature, showing an evolution from a paramagnetic (at low temperature) to a diamagnetic behavior (at room temperature). Further, the room temperature magnetic properties indicate a ferromagnetic order even for the un-doped ZnO film. The results open new perspectives for the development of multifunctional ZnO semiconductors, the GLAD co-sputtering technique enables the control of the magnetic response, even in the un-doped semiconductor materials.

## 1. Introduction

Ferromagnetic properties in semiconductors are an exciting field of research to integrate electrical and magnetic functionalities in nanostructured materials. In the last decade, different works have addressed the mechanisms responsible for the room temperature ferromagnetism (RTFM) in the so-called diluted magnetic semiconductors (DMS), where a semiconductor as ZnO is doped by ferromagnetic transition metal (Mn, Fe, Co, Ni) [1,2,3,4,5]. In particular, some hypotheses on the origin of the ferromagnetism in these materials have been raised. For instance, the segregation of metallic cluster [6] and double exchange [3,4] are some of the effects that can be responsible for the room temperature ferromagnetism (RTFM). At the same time, Coey et al. [5] proposed that the ferromagnetic exchange in DMS materials is related to bound magnetic polarons (BMPs) mediated with shallow donor electrons. In addition, distinct authors related the RTFM properties with structural defects, in particular when metal dopants are employed [5,7].

However, some recent works have brought to light the RTFM in non-magnetic metal-doped ZnO, such as ZnO:Cu [8] and ZnO:Ag [9]. In these systems, the existence of the RTFM is associated with BMPs [5] and defects due to Zn or O vacancies. In this context, the ZnO and metal-doped ZnO have been explored in distinct samples geometries. For nanoparticles (powder), the magnetic properties are studied in a wide range of temperatures, including room temperature [1,8,9,10,11]. On the other hand, considering the film geometry, the deposition technique, the deposition parameters and thicknesses of the films can be taken to advantage to change not just the structural properties of the ZnO and/or metal-doped ZnO, but also the magnetic properties, leading to interesting multifunctional materials for technological applications [7,12,13].

The ferromagnetic response of ZnO has been optimized by employing distinct deposition parameters, metal dopant concentrations, and annealing procedures. However, there is a lack of reports showing the modification of the magnetic properties of ZnO and metal-doped ZnO material through the change of the columnar growth of the films. In this context, the glancing angle deposition (GLAD) co-sputtering technique arises as a powerful tool to induce zigzag architecture in the columnar growth of the films [14,15]. These features, in turn, can be used to lead to the formation of magnetic moments related to defects in the ZnO structure.

In this work, the magnetic properties of columnar zigzag-like ZnO and Ag-doped ZnO films have been addressed. The films have been prepared by GLAD co-sputtering and the properties of the films have been correlated with the preparation conditions. In addition, an annealing protocol was used to modify the structures of the ZnO and Ag-ZnO films. Our results open new perspectives for the multifunctionalization of ZnO semiconductors, where the GLAD technique allows to tune the magnetic response even in un-doped semiconductor materials.

## 2. Materials and Methods

### 2.1. Films Deposition

**Figure 1:**
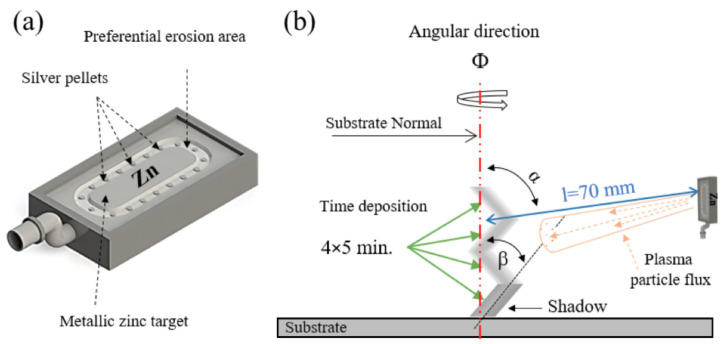
(**a**) Schematic representation of the distribution of the Ag pellets on the Zn target and (**b**) illustration of the GLAD system, where Φ is the angular direction, α is the incidence angle of the Zn particle flux and β is the columnar growth angle.

ZnO and Ag-doped ZnO thin films were DC sputtered from a metallic zinc (Zn) target (with dimensions of 20×10×0.6 cm${}^{3}$ and 99.96 at.% purity), using a custom-made vacuum chamber. The Zn target was customized with different amounts of Ag pellets (with individual area of ≈0.2 cm${}^{2}$), symmetrically distributed along the preferential erosion area (≈50 cm${}^{2}$) [16], to tune the silver content in the coatings. Figure 1 presents a schematic representation of the experimental set-up used in the preparation of the thin films. The incidence angle of the Zn particle flux (α) was determined from the substrate normal, by tilting the substrate holder at $45^{\circ }$ [17]. The Zn target was sputtered during 20 min, 4×5 min for each chevron component of the zigzag structure, as illustrated in Figure 1b. Here, a constant argon flow rate of 25 sccm and a total pressure of 0.38 Pa was employed. Oxygen was added to the gas atmosphere as reactive gas, at a flow rate of 20 sccm, corresponding to a partial pressure value of 0.2 Pa. The films were prepared at room temperature, and the experimental conditions and sputtering time were set to keep the overall thickness close to 1μm for all films. A plasma reactor (Zepto) was used to sputter-clean the substrates (glass ISO norm 8037-1 microscope slides, (100) p-type silicon wafers and biaxial oriented Polyethylene Terephthalate from Goodfellow), considering pure Ar atmosphere and RF power of 100 W during 900 s. Specifically, the set of produced samples consist of pure ZnO film and Ag-doped ZnO films with intermediate (10 at.% Ag-ZnO) and high (20 at.% Ag-ZnO) Ag concentrations: The films were cut into three pieces. While one of this was kept as-cast, the other two pieces were annealed at 523 and 673 K for 60 min, with heating and cooling rates of 10 K/min.

### 2.2. Structural and Magnetic Characterization

The structural properties were obtained through X-ray diffraction (XRD) (Cu-Kα radiation) by using a Rigaku Miniflex II diffractometer in the Bragg-Brentano (θ-2θ) geometry. The morphology and thickness of the ZnO thin films were characterized by Scanning Electron Microscopy (SEM), using a NanoSEM - FEI Nova 200 (FEG/SEM) microscope, on fractured cross-sections and top views conditions. At the same time, Energy Dispersive X-ray Spectroscopy (EDAX - Pegasus X4M (EDS/EBSD) was used to evaluate the O, Zn and Ag content in the films. Raman experiments were conducted in a confocal microscope spectrometer (LabRAM HR Evolution) with a 100× objective lens in the 300–800 cm${}^{-1}$ spectral range for a 532 nm (2.33 eV) laser line. The measurements were conducted at room temperature in backscattering geometry and with a 1800 g mm${}^{-1}$ diffraction grating. The laser power was kept low to avoid sample heating.

Regarding the magnetic characterization, Electron Paramagnetic Resonance (EPR) experiments were performed using a Bruker EPR system operating at 9.89 GHz (TE011 mode). The samples were placed near the center of a cylindrical cavity, where the microwave magnetic field was maximized. The external magnetic field, applied in the film plane, varied from 1.0 up to 4.0 kOe, modulated at an amplitude of 0.1 Oe at 100 kHz. The magnetic properties were further evaluated with a Quantum Design Dynacool Physical Property Measurement System (PPMS) and a Lake Shore model 7404 vibrating sample magnetometer (VSM). In the first case, isothermal magnetization curves at 5, 50, 100, 200, and 300 K were obtained. Moreover, the zero-field-cooled (ZFC) and field-cooled (FC) magnetization curves, acquired in the temperature range between 5 and 300 K and under a constant field of 200 Oe, were investigated. Considering the low magnetic moment observed in the PPMS results, the RTFM behavior of the films was also verified through VSM measurements.

## 3. Results and Discussion

### 3.1. Structural Properties

**Figure 2:**
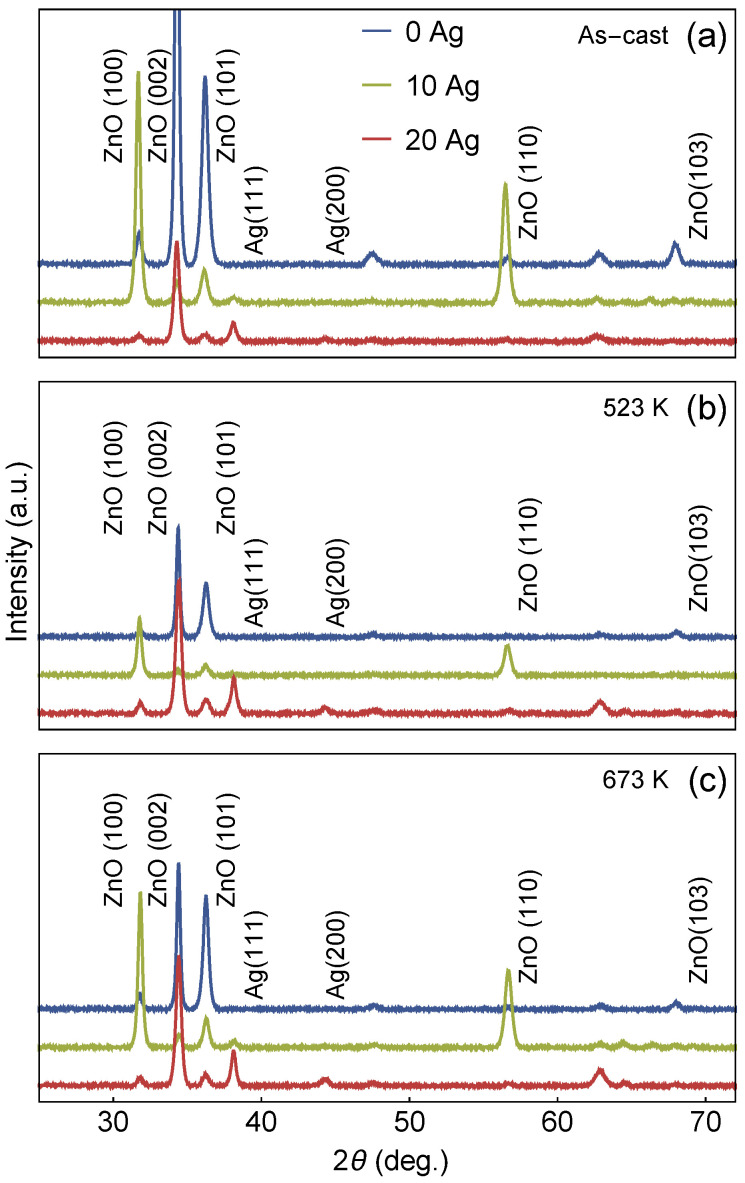
XRD diffractograms for the studied samples. (**a**) X-ray diffraction for the as-cast films with different Ag concentration. Similar plot for the films annealed at (**b**) 523 K and (**c**) 673 K.

Figure 2a–c shows the XRD patterns of the ZnO and Ag-doped ZnO films. The XRD patterns reveal phases of ZnO, with hexagonal structure and P63mc space group (ICSD-26170), and of Ag, with a face-centered cubic structure (ICSD-44387). Independently of the annealing temperature, the planes (100), (002), (101), and (110) are identified to the ZnO phase, while the planes (111), and (200) are associated to the Ag one. Remarkably, all the samples show the [002] direction as the preferred growth orientation. This corresponds to the assessment that the aforementioned plane is thermodynamically favorable in such systems [18]. In fact, similar results have been already reported [19], attributing the preferred growth orientation to changes in the lattice constant of the ZnO nanoparticles. For the pure ZnO films, i.e., the samples without any Ag doping, the Rietiveld refinements reveal an increase of the particle size with increasing annealing temperature. For the as-cast films, the average particle size is around 29 nm, whereas, for the films annealed at 673 K, the average particle size increases to around 37 nm.

A change in lattice parameters with increasing Ag content is also observed for all samples. For example, pure ZnO presents a lattice parameter of round 3.2328 with a *c* value of around 5.0055 in the as-cast samples. However, for 20% of Ag dopant the lattice parameter and *c* values are 3.2720 and 5.2425, respectively. Further, not just the Ag-dopant influences the lattice parameter variations, but also the annealing temperature. For instance, for the 20% Ag-dopant sample the lattice parameter *c* increases from 5.2425 (as-cast) to 5.2627 when annealed at 673 K, evidencing that the annealing process increases the c-axis values, most probably based on the ZnO lattice distortions induced by the addition of Ag [20].

This distortion is evidenced by the XRD diffractograms, where the addition of Ag content leads to modifications of the space group and structure of the films. For the 10 at.% Ag-ZnO film, the diffractograms disclose the preferential planes of (100) and (110). This feature is related to the change of the space group in the ZnO. Specifically, the 10 at.% Ag-ZnO films present the P6122 space group, whereas for the 20 at.% Ag-ZnO films, a tetragonal structure, characterized by the preferential orientation planes (002) and (103) with I4cm space group, is observed. These changes in the space group are due to the insertion of Ag in the lattice of the ZnO films and, as a consequence, leading to instability in the hexagonal structure, which indicates a formation of strain in these samples. Zhen Fan et al. observed that in BiFeO${}_{3}$ films, there is favoritism in the (110) plane due to the strain and, consequently, a change in the spatial group of Pmc$2_{1}$ to the C*c*/Ima*2* [21]. Christen et al. also observed that the strain in the BiFeO${}_{3}$ thin films causes a change in the crystalline structure, in which the samples change from rhombohedral to tetragonal structure [22]. The strain in the samples was calculated by the standard procedure given by

(1) $$\beta_{hkl}cos\left(\theta \right)=(\frac{k\lambda }{D})(\frac{4\sigma \sin(\theta )}{Y_{hkl}}),$$

where $\beta_{hkl}$ is the peak width at half-maximum intensity, θ is peak position, λ is the wavelength of the radiation, k=0.94, σ is the stress and $Y_{hkl}$ is the Young’s modulus [23,24]. The strain values, as well as the main features obtained from the Rietveld refinement, are summarized in Table 1. In particular, the results are in good agreement with values reported in the literature [23].

**Figure 3:**
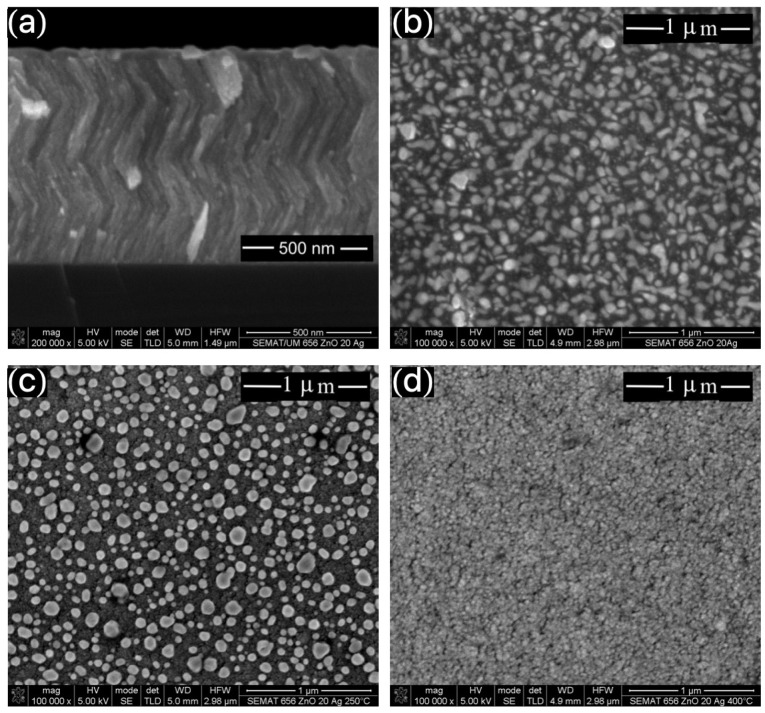
Representative SEM micrograph images for the 20 at.% Ag-ZnO films (**a**) Cross-section image for the as-casted film, where the zigzag-like architecture is evidenced. Surface SEM image for an (**b**) as-cast film, (**c**) film annealed at 523 K, and (**d**) film annealed at 673 K.

Figure 3a shows representative SEM cross-section image of the 20 at.% Ag-ZnO film, in which a clear zigzag-like architecture is observed. In particular, all samples present similar behavior, independently of the Ag presence or content, and annealing temperature. The results are in agreement with previous reports employing similar technique [17]. Figure 3b–d shows the SEM micrographs obtained for the 20 at.% Ag-ZnO films annealed at different temperatures. For the as-cast film (Figure 3b), the Ag clusters are dispersed in the ZnO film, a feature that is strongly modified as the annealing temperature increases. For the films annealed at 523 K (Figure 3c), the Ag clusters migrate to the film surface, keeping the size unchanged, when compared with the as-cast film. However, for the film annealed at 673 K (Figure 3d), the annealing temperature is high enough to modify the Ag cluster size, yielding an Ag layer under the film surface. Besides, the employed temperatures during the annealing are not high enough to achieve the sintering of the films.

**Figure 4:**
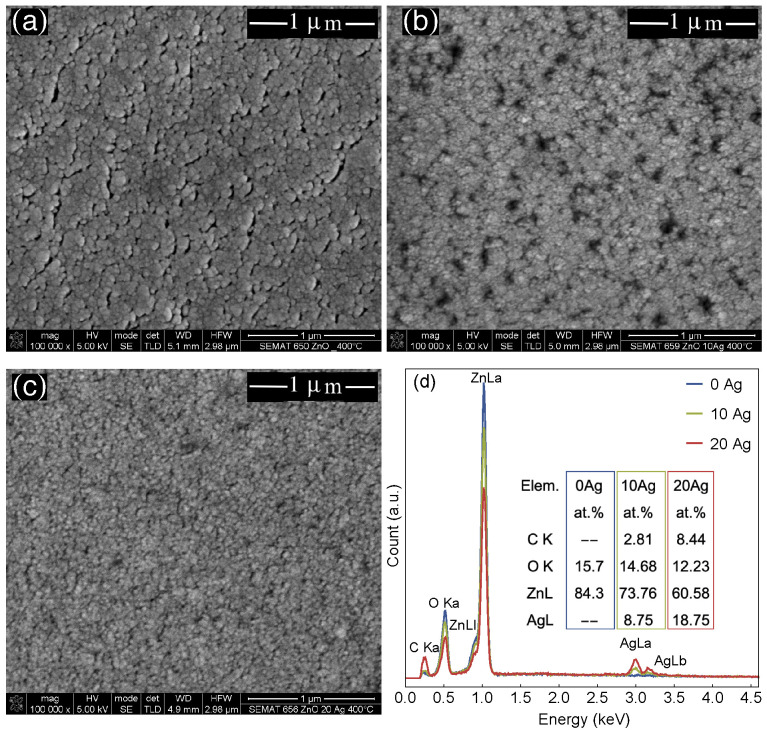
Representative SEM micrograph images for the films annealed at 673 K, (**a**) Pure ZnO film, (**b**) 10 at.% Ag-ZnO film, and (**c**) 20 at.% Ag-ZnO (Note it is similar to that one presented in Figure 3d). (**d**) EDS results for the studied films, the inset indicating the at.% values for the different Ag concentrations.

Figure 4a–c shows the SEM micrograph images for the films with distinct Ag contents annealed at 673 K. As the annealing temperature increases, the migration of the Ag clusters to the surface of the film leads to important modifications, resulting in an Ag layer covering the ZnO films. The 10 at.% Ag-ZnO films present large roughness due to the size of the Ag clusters. On the other hand, for the 20 at.% Ag-ZnO films, the Ag layer shows a slight decrease in roughness, related to the number and proximity of the Ag particles, facilitating the percolation of the clusters.

At last, Figure 4d shows the EDS results for the ZnO and Ag-doped ZnO films without annealing, also showing the at.% and Ag concentration values of 8.75% and 18.75% obtained for the 10 at.% Ag-ZnO and 20 at.% Ag-ZnO films, respectively.

**Figure 5:**
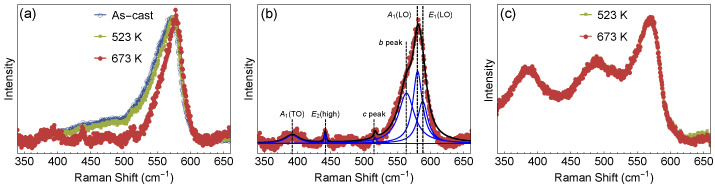
Raman spectra of (**a**) the as-cast ZnO film and annealed films at 573 K and 673 K, respectively. (**b**) The Raman spectrum of the sample annealed at 673 K is fitted to a sum of Lorentzians (blue curves) and shows the characteristic peaks of the asymmetric band centered at about 575 cm${}^{-1}$. (**c**) Raman spectra of the Ag-doped ZnO for 20 at.% Ag-ZnO annealed at 573 K and 673 K, respectively. The spectra were normalized by the maximum peak for better visualization.

Raman experiments have been carried out to further investigate the effect of annealing temperature and Ag doping on the ZnO films. Figure 5a shows the Raman spectra of the as-cast ZnO films and annealed at 523 K and 673 K, respectively. It can be observed an asymmetric band centered at about 575 cm${}^{-1}$ which is due to the convolution of multiple phonons across the Brillouin zone.

In order to estimate the spectral position of these different contributions, a fitting procedure of this band was carried out using a sum of Lorentzian curves, as shown in Figure 5b. This asymmetric band was fitted by three peaks located at 558 cm${}^{-1}$ (named as b peak), 575 cm${}^{-1}$ and 583 cm${}^{-1}$, respectively (see Figure 5b). There is also a peak at about 510 cm${}^{-1}$ identified as c peak. The two peaks at around 575 and 583 cm${}^{-1}$ are associated with the A${}_{1}$(LO) and E${}_{1}$(LO) phonon modes, respectively [25,26]. The presence of the E${}_{1}$(LO) indicates that the samples present lattice defects (oxygen vacancy and zinc interstitial) [26,27,28]. The appearance of the b and c peaks may also be associated with lattice defects since it breaks the crystal lattice symmetry, which ensures momentum conservation of these new Raman features. In addition, it is also noticed peaks at about 380 cm${}^{-1}$ and 437 cm${}^{-1}$, respectively, associated with the A${}_{1}$(TO) and E${}_{2}$(high) phonon modes [25,26]. The A${}_{1}$(LO) and E${}_{2}$ phonon modes are characteristics Raman fingerprints of the ZnO wurtzite phase [25,27,29]. The fitting procedure has been presented for the sample annealed at 673 K, being representative for the rest of the samples.

However, it is to notice that for the 573 K and as-cast samples, the asymmetric band and the E${}_{2}$(high) phonon mode become broader (see Figure 5a). The E${}_{2}$ phonon mode is associated with the vibration of the oxygen atoms [25], thus the observed broadening may be occurring by changes of the oxygen atoms within the lattice due to the annealing. Raman spectra of the Ag-doped ZnO films for 20 at.% Ag-ZnO annealed at 573 K and 673 K is shown in Figure 5c, which are superimposed to each other. Both spectra present similar behavior and two new bands located at about 381 and 489 cm${}^{-1}$ are observed. Besides, it is worth noting that the frequency position of the characteristic peak of the ZnO film at 575 cm${}^{-1}$ (see Figure 5b) decreases to 568 cm${}^{-1}$ in the Raman spectrum of the Ag-doped ZnO film. The appearance of these two new bands and peak shift are related to lattice modification of the ZnO due to the addition of Ag dopant, this is in agreement with XRD analysis where a change from P63mc to P6122 space group is observed.

### 3.2. Magnetic Results

**Figure 6:**
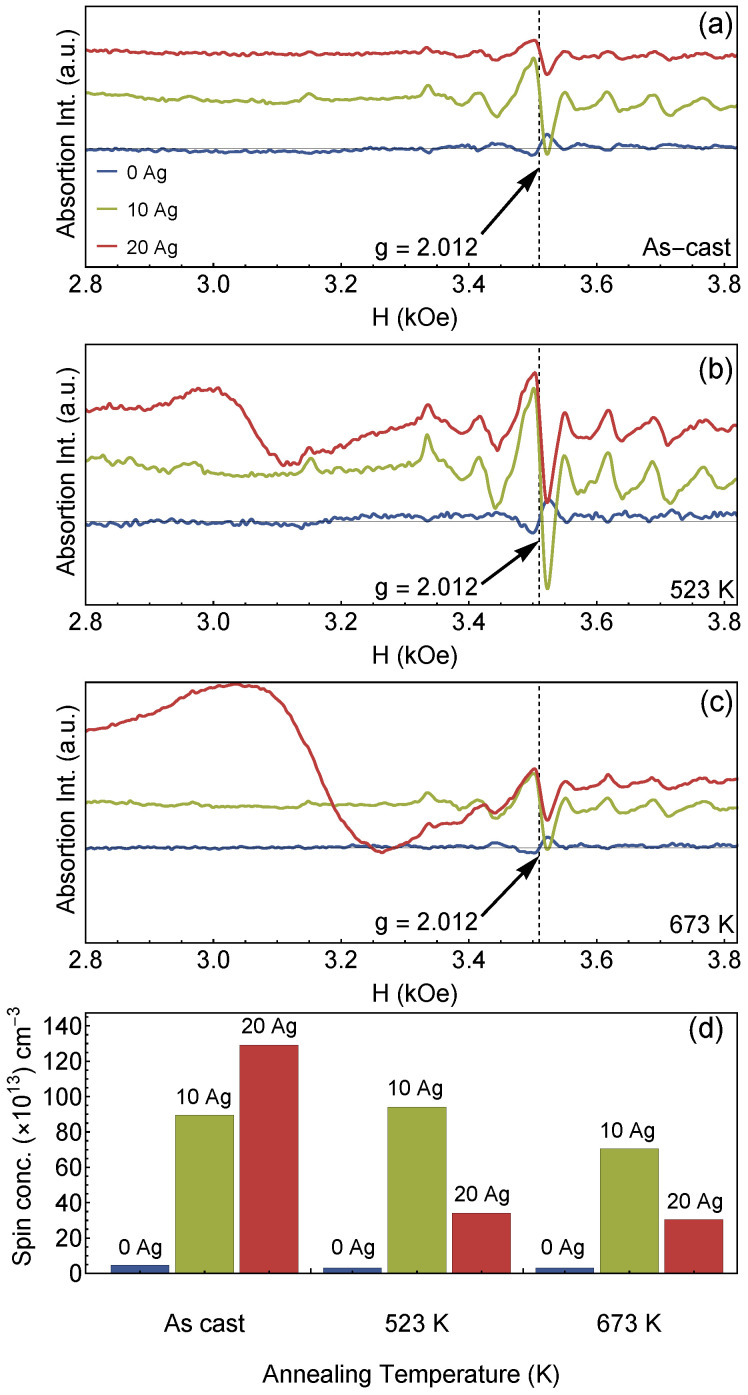
EPR spectra of the ZnO and Ag-doped ZnO films: spectrum for the (**a**) as-cast films, and films annealed at (**b**) 523 K and (**c**) 673 K. In particular, the g = 2.012 enables to infer that the samples present Zn or O vacancy sites, which may yield RTFM behavior. (**d**) Spin concentration calculated from the EPR spectrum.

Figure 6 shows the EPR measurements for the different films studied in this work. It can be observed a remarkable resonance peak at around g=2.012, disclosing the Zn vacancy in the films [8,30]. The pure ZnO films show a low-intensity absorption peak, while the 10 at.% Ag-ZnO films show a larger absorption peak intensity, which increases considerably, independently of the annealing temperature. The spin concentration (N) can be estimated through the relation [8] $N\propto I{\left(\Delta H_{pp}\right)}^{2}$, where *I* is the intensity, and $\Delta H_{pp}$ is the peak-to-peak width of the EPR spectrum.

Figure 6d shows the EPR results of the samples showing a considerable enhancement in the spin concentration when Ag is inserted in the structure. At the same time, the increase of the annealing temperature leads to a decrease in spin concentration for the doped films. This behavior is associated with the migration of the Ag cluster to the surface of the film, suppressing the spin concentration contributing to the EPR signal. In addition, the increase of the vacancy sites is also a result of the employed deposition technique and the zigzag architecture in films. As previously discussed, the room-temperature ferromagnetism in semiconducting systems can be induced through, for instance, Zn or O vacancies, segregation of metallic clusters, as well as BMPs [3,4,5,6,7].

**Figure 7:**
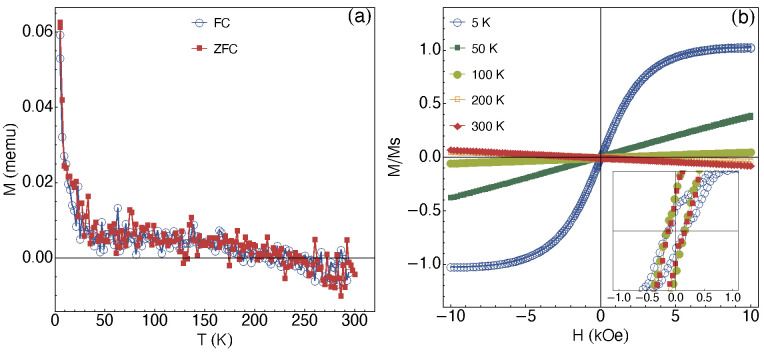
(**a**) ZFC-FC magnetization curves for the 10 at.% Ag-ZnO films annealed at 523 K. The crossover of the magnetic moment from positive to negative values at around 190 K is verified. (**b**) Representative isothermal magnetization curves. The inset shows a detailed view of the curves at the low-field range.

It is important to point out that all films studied in this work present the same ZnO “*matrix*”, irrespective of the Ag content. Therefore, it is expected that the magnetic behavior of ZnO dominates the magnetic response, i.e., the paramagnetic/diamagnetic and ferromagnetic (if it exists) features that may overlap. Magnetic measurements were performed in a wide range of temperatures and fields, as presented in Figure 7. The ZnO “matrix” represents the main contribution to the magnetic signal, leading to similar results independently of the studied films. However, after the removal of the paramagnetic/diamagnetic signal, it is confirmed a ferromagnetism behavior.

Figure 7a shows the representative ZFC-FC magnetization curves for the 10 at.% Ag-ZnO film annealed at 523 K, in which a dependence with temperature is observed. At the same time, it is possible to observe a transition temperature, in which the magnetic moment crosses over zero, leading to a diamagnetic behavior for temperatures above 190 K.

All these features are revealed in the isothermal magnetization curves, shown in Figure 7b were the transition from a paramagnetic behavior to a diamagnetic one is verified. The curves acquired at 5, 50, and 100 K, respectively, uncover a predominant paramagnetic behavior in the films. On the other hand, for the curves measured at 200 K and 300 K suggests the diamagnetic behavior. The inset in Figure 7b shows in detail these curves for the low-field range, after the remotion of the dia/paramagnetic contribution from the signal of the curves. From these curves, we can observe a weak ferromagnetic behavior. In particular, similar features for the isothermal magnetization curves have already been observed for nanoparticles composed by Ag-doped ZnO and V-doped ZnO alloy [11,31].

**Figure 8:**
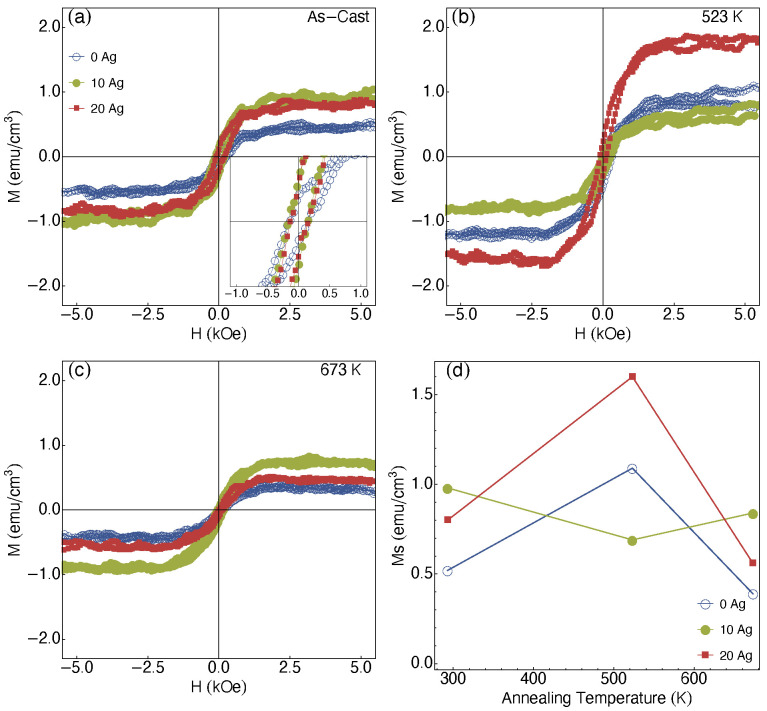
Magnetization curves measured at room temperature for distinct Ag concentrations for the (**a**) as-cast films, and films annealed at (**b**) 523 K and (**c**) 673 K. (**d**) Magnetization saturation for films with distinct Ag concentration and as a function of the annealing temperature.

Figure 8 shows the magnetization curves measured at room temperature for all films. In particular, the diamagnetic signal due to the ZnO “matrix” and the sample holder signal are subtracted from the experimental data; therefore, only the ferromagnetic contribution is shown. For the as-cast ZnO and Ag-doped ZnO films, it is verified a hysteretic behavior with a significant coercive field, a fact that can be confirmed through the inset in Figure 8a.

The as-cast films reach magnetization saturation (M${}_{s}$) of 0.98 emu/cm${}^{3}$ for the 10 at.% Ag-ZnO, decreasing to 0.8 emu/cm${}^{3}$ and 0.6 emu/cm${}^{3}$ for pure ZnO and 20 at.% Ag-ZnO films, respectively. However, for the films annealed at 523 K, shown in Figure 8b, an increase in the M${}_{s}$ is observed for the pure ZnO and 20 at.% Ag-ZnO films, reaching values of around 1.1 emu/cm${}^{3}$ and 1.6 emu/cm${}^{3}$, respectively. On the other hand, the 10 at.% Ag-ZnO film shows a slight decrease in the M${}_{s}$ reaching 0.69 emu/cm${}^{3}$. Finally, for the films annealed at 673 K (Figure 8c), it is verified a substantial decrease in the M${}_{s}$ for the pure ZnO and 20 at.% Ag-ZnO films, while the M${}_{s}$ observed for the 10 at.% Ag-ZnO film increases slightly.

Figure 8d shows the saturation magnetization as a function of the annealing temperature for the ZnO and Ag-doped ZnO film. Considering the pure ZnO films, we verified M${}_{s}$ values between 0.4 and 1.0 emu/cm${}^{3}$. For these samples, the RTFM is associated with defects in the columnar growth, favored by the zigzag-like architecture induced during the film deposition. This last, in its turn, is responsible for generating structural defects resulting in the RTFM [5,7]. In particular, the changes of the M${}_{s}$ as a function of annealing can be associated to the changes structural behavior, observed by XRD and Raman analysis. Regarding to Ag-doped ZnO films, besides the zigzag-like architecture, the Ag substitution, as well as the migration of the Ag cluster in the films contribute to the RTFM in our films.

For the Ag-doped films, we verified an overlap of distinct contributions for the RTFM behavior. In these cases, besides of the zigzag-like architecture, the substitutions induced by the Ag clusters contribute to the M${}_{s}$ variation. While the saturation magnetization slightly decreases for the 10 at.% Ag-ZnO films as the annealing increases, for the 20 at.% Ag-ZnO films we observe an increase and successive decrease in the M${}_{s}$ as a function of the annealing temperature. In particular, for 20 at.% Ag-ZnO films, it is associated to a dynamics of the sample’s defects, reducing the overlapping of the BMPs [8], thus leading to a reduction of the M${}_{s}$. Our results disclose the findings already reported in Ref. [32], where a systematic study of the ferromagnetism induction on ZnO films using different non-magnetic metals is presented, in which a 10 nm-thick layer was deposited onto the ZnO film, and where no magnetic response for the as-cast samples is observed. Similar results are observed for the pure ZnO material [1,11,32].

These results indicate that the control of the columnar growth of the films (zigzag, for instance) is as efficient as to doping semiconductor materials with metals. Therefore, our findings open a new perspective to produce semiconductor materials functionalized with RTFM behavior for application in spin coupled optoelectronic devices, among others.

## 4. Conclusions

It is shown that the GLAD co-sputtering technique can be used as a powerful tool to induce zigzag-like architecture in ZnO and Ag-doped ZnO films. The overlap between the employed deposition strategy and the Ag insertion in the ZnO films led to tune the room temperature ferromagnetism response, which is observed even in the un-doped film. At the same time, the annealing process enables to manipulate the Ag clusters and the oxygen vacancies in the ZnO films. As a consequence, the ferromagnetic moment shows a substantial increase for the samples annealed at 523 K. The magnetic results are explained by the structural, morphological and dynamic results obtained from the XRD, SEM and EPR experiments. Thus, the present results open new perspectives for the multi-functionalization of ZnO semiconductors, where the GLAD co-sputtering allows us to control the induction of ferromagnetic moment even in un-doped semiconductors materials.

## Figures and Tables

**Table 1:** Parameters obtained from the Rietveld refinement for the studied films. Here, the size and the strain are indicated in nm and MPa, respectively.

Annealing	Film	a = b (Å)	c (Å)	Size	Strain
As-cast	Pure ZnO	3.2328	5.0055	29	110
	10 at.% Ag-ZnO	3.2520	5.0166	33	135
	20 at.% Ag-ZnO	3.2720	5.2425	36	165
523 K	Pure ZnO	3.2330	5.0132	32	117
	10 at.% Ag-ZnO	3.2525	5.0766	37	140
	20 at.% Ag-ZnO	3.2728	5.2555	38	155
673 K	Pure ZnO	3.2337	5.1519	37	123
	10 at.% Ag-ZnO	3.2528	5.2118	40	147
	20 at.% Ag-ZnO	3.2729	5.2627	42	168
